# The effect of *Abi3* locus deletion on the progression of Alzheimer’s disease-related pathologies

**DOI:** 10.3389/fimmu.2023.1102530

**Published:** 2023-02-21

**Authors:** Hande Karahan, Daniel C. Smith, Byungwook Kim, Brianne McCord, Jordan Mantor, Sutha K. John, Md Mamun Al-Amin, Luke C. Dabin, Jungsu Kim

**Affiliations:** ^1^ Stark Neurosciences Research Institute, Indiana University School of Medicine, Indianapolis, IN, United States; ^2^ Department of Medical and Molecular Genetics, Indiana University School of Medicine, Indianapolis, IN, United States; ^3^ Medical Neuroscience Graduate Program, Indiana University School of Medicine, Indianapolis, IN, United States

**Keywords:** ABI3, Alzheimer’s disease, microglia, inflammation, 5xFAD

## Abstract

Human genetics studies of Alzheimer’s disease (AD) have identified the *ABI3* gene as a candidate risk gene for AD. Because *ABI3* is highly expressed in microglia, the brain’s immune cells, it was suggested that ABI3 might impact AD pathogenesis by regulating the immune response. Recent studies suggest that microglia have multifaceted roles in AD. Their immune response and phagocytosis functions can have beneficial effects in the early stages of AD by clearing up amyloid-beta (Aβ) plaques. However, they can be harmful at later stages due to their continuous inflammatory response. Therefore, it is important to understand the role of genes in microglia functions and their impact on AD pathologies along the progression of the disease. To determine the role of ABI3 at the early stage of amyloid pathology, we crossed *Abi3* knock-out mice with the 5XFAD Aβ-amyloidosis mouse model and aged them until 4.5-month-old. Here, we demonstrate that deletion of the *Abi3* locus increased Aβ plaque deposition, while there was no significant change in microgliosis and astrogliosis. Transcriptomic analysis indicates alterations in the expression of immune genes, such as *Tyrobp*, *Fcer1g*, and *C1qa*. In addition to the transcriptomic changes, we found elevated cytokine protein levels in *Abi3* knock-out mouse brains, strengthening the role of ABI3 in neuroinflammation. These findings suggest that loss of ABI3 function may exacerbate AD progression by increasing Aβ accumulation and inflammation starting from earlier stages of the pathology.

## Introduction

Human genetic studies of Alzheimer’s disease (AD) have identified many risk variants in the loci harboring microglia-enriched genes or microglia-specific enhancers (1–3). Importantly, pathway analyses of common and rare AD risk variants converge on immune response, phagocytosis, and lipid metabolism (4, 5). These biological processes are mainly regulated by microglia in the brain, strengthening the critical role of these cells in AD. Gliosis, including microglial activation, has been considered one of the pathological hallmarks of the disease after amyloid plaques and neurofibrillary tangles. Many studies have demonstrated that microglia become activated starting from the very early stages of AD, and this can result in beneficial or detrimental effects throughout the course of the disease. Microglia are the primary immune cells of the brain, secrete proinflammatory cytokines in response to toxic stimuli, such as amyloid-beta (Aβ) and apoptotic cells, and phagocytose them (6, 7). However, these beneficial effects can be overshadowed by a prolonged microglial inflammatory response. This may exacerbate neurodegeneration at later stages of the disease. Functional studies with microglial AD risk genes also demonstrated that the deletion of these genes could affect AD-related pathologies in different ways, sometimes even in opposite directions (8–14). These studies have led to the notion that microglia can have differential effects on AD pathology depending on the disease stage. Therefore, it is imperative to assess the effects of genetic and pharmacological manipulations of microglial genes on AD-related pathologies at different stages of the disease.

Recent human genetics studies of AD identified a risk variant in the Abelson interactor family member 3 (*ABI3*) locus in late-onset AD (LOAD) patients (2, 15–17). ABI3 is a microglia-enriched gene (16, 18, 19). It participates in the WASP-family verprolin homologous protein (WAVE) regulatory complex, which is involved in actin cytoskeleton organization (20, 21). In our earlier study, we investigated the effect of *Abi3* locus deletion on AD-related pathologies by using 8-month-old 5XFAD mice (22). We demonstrated that the loss of ABI3 function dramatically increased Aβ accumulation and exacerbated neuroinflammation and synaptic dysfunction in these mice. However, a recent study, using the TgCRND8 transgenic mouse model, demonstrated that deletion of the *Abi3* locus reduced Aβ levels in 3-month-old mice, whereas this effect was diminished in 6-month-old mice (23). These data highlight the importance and necessity of a thorough assessment of the pathological changes during disease progression. This is especially critical for neurodegenerative diseases, where earlier therapeutic interventions have a higher potential to impact the disease progression before neurodegeneration occurs.

In this brief report, we assessed the effects of *Abi3* locus deletion on AD-related pathologies using 4.5-month-old 5XFAD mice to gain better insight into how ABI3 may play a role in AD progression. Considering the differential effects of microglia functions on AD-related pathologies during disease progression, we hypothesized that modulation of ABI3 may affect these pathological changes differently at earlier stages of the disease. Here, we demonstrated that deletion of the *Abi3* locus increases Aβ deposition in 4.5-month-old 5XFAD mice. We found upregulation of immune genes in *Abi3* knock-out mice similar to the older cohort. However, we also identified marked transcriptomic differences between the young and old cohorts, underlining the divergent effects of microglial genes during disease progression. Furthermore, we identified a significant increase in several inflammatory cytokines in *Abi3* knock-out mice in this young cohort. Overall, these data demonstrate that loss of ABI3 function increases neuroinflammation and Aβ accumulation in 5XFAD mice starting from earlier ages.

## Materials and methods

### Animals

5XFAD mice were purchased from The Jackson Laboratory [MMRRC 34840, B6SJL-Tg(APPSwFlLon,PSEN1*M146L*L286V)6799Vas/Mmjax)], and *Abi3* knock-out mice were obtained from the MMRRC [C57BL/6N-Abi3tm1.1(KOMP)Vlcg]. These mice were crossbred to generate 5XFAD mice expressing two copies of *Abi3* (*5XFAD;Abi3^+/+^*, referred to as *Abi3^+/+^*) and no copies of *Abi3* (*5XFAD;Abi3^-/-^*, referred to as *Abi3^-/-^*). Female 4.5-month-old *Abi3^+/+^* and *Abi3^-/-^* mice were used in the experiments. Mice were housed under standard conditions with free access to food and water. All animal experiments were approved and performed in compliance with the guidelines of the Institutional Animal Care and Use Committee at Indiana University.

### Tissue collection and sample preparation

Mice were anesthetized with Avertin and perfused with PBS. Brains were removed and separated into two hemispheres. The right hemisphere was used for biochemical experiments. The left hemisphere was fixed in 4% paraformaldehyde to be used in histology experiments. Tissue samples were embedded in paraffin and sectioned at 5µm thickness at the Histology and Histomorphometry Core.

### Western blotting

Sequential protein extraction was performed on the cortical tissues with PBS, RIPA, and Guanidine buffer in the presence of protease and phosphatase inhibitors, as described previously (24). PBS extraction was performed gently using a hand-held grinder. Equal amounts of proteins (10µg) from RIPA fractions were loaded onto 4-20% TGX gels (Bio-Rad), separated by gel electrophoresis, and transferred onto polyvinylidene difluoride membranes. Blots were probed with the antibodies against amyloid precursor protein (APP) (Invitrogen, 51-2700), β-secretase 1 (BACE-1) (Cell Signaling Technology, 5606), Aβ 82E1 (IBL-AMERICA, IBL10323), insulin-degrading enzyme (IDE) (Abcam, ab32216), neprilysin (NEP) (Abcam, 208778), and β-actin (Sigma, A1978). Signals were visualized by chemiluminescence. Blots were quantified with ImageJ. Results were normalized by β-actin levels and presented as a fold change relative to the *Abi3^+/+^* genotype.

### Electrochemiluminescence assay for Aβ and cytokine

To measure Aβ40 and Aβ42 levels, V-PLEX Plus Aβ Peptide Panel (6E10) Kit (K15200E, Meso Scale Discovery, MSD) was used following the manufacturer’s instructions. Aβ40 and Aβ42 levels were measured in the PBS, RIPA, and guanidine extracts of the brain samples using the MESO QuickPlex SQ120 (MSD). The concentrations were normalized by total protein levels in the samples.

For cytokine measurement, the V-PLEX Cytokine Panel Mouse Kit (K15245D, MSD) was used. PBS and RIPA-soluble extracts from mouse cortices were loaded into the panel to measure the levels of 9 cytokines. We were able to detect 5 of them in the brain samples. The concentrations were normalized by total protein levels in the samples.

### Immunohistochemistry

Coronal sections were deparaffinized, and antigen retrieval was performed with citrate buffer in a 70 °C heated water bath. We stained the sections with X-34 dye to detect fibrillar amyloid plaques, as described previously (22). For immunofluorescence staining, sections were blocked with PBS containing 5% normal donkey or goat serum. The slides were then incubated with anti-IBA1 (WAKO, 013-27691) or GFAP (Invitrogen, 13-0300) antibodies, followed by incubation with Alexa Fluor 568–goat anti-rabbit (Invitrogen, A11036) or Alexa Fluor 488–donkey anti-rat (Jackson ImmunoResearch, 712545150) antibodies. Sections were mounted on slides with Aqua-Poly/Mount mounting medium.

### Image analyses

Images were captured using an inverted fluorescence microscope (DM IRB, Leica Biosystems). Staining was quantified in cortical regions using ImageJ (25). The average of three sections from different anatomical coordinates (150 µm distant) was used to quantify the area covered by X34+ plaques, IBA1+, and GFAP+ cells for each mouse. The number of plaques was normalized by the total area analyzed. For colocalization analysis, Ilastik v1.3.3 (26) was used to classify pixels into IBA1+ cells, GFAP+ cells, and X34+ plaques. Ilastik produced probability maps of the classifications. These classified probability maps were then imported to CellProfiler v4.2.5 (27) to quantify the percent area colocalized by X34+ plaques and IBA1+ or GFAP+ cells.

### NanoString analyses

Total RNA was extracted from cortical tissues using TRIzol (MRC). The NanoString Mouse AD gene expression panel was used for gene expression profiling on the nCounter platform, as described by the manufacturer. The data were analyzed using the nSolver Analysis Software 4.0 (NanoString). Pathway, gene ontology, and network analyses were performed using the MetaCore™ software.

### Statistical analyses

Statistical analyses were performed using GraphPad Prism 8 (GraphPad Software). The unpaired two-tailed t-test was used for the comparison of the two groups. The correlation analyses between Aβ40 and Aβ42 levels were performed using Pearson’s correlation test. Data were represented as mean ± SEM. Sample sizes and statistical analyses for each experiment were indicated in figure legends.

## Results

### Amyloid-β accumulation in *Abi3* knock-out mice

**Figure 1:**
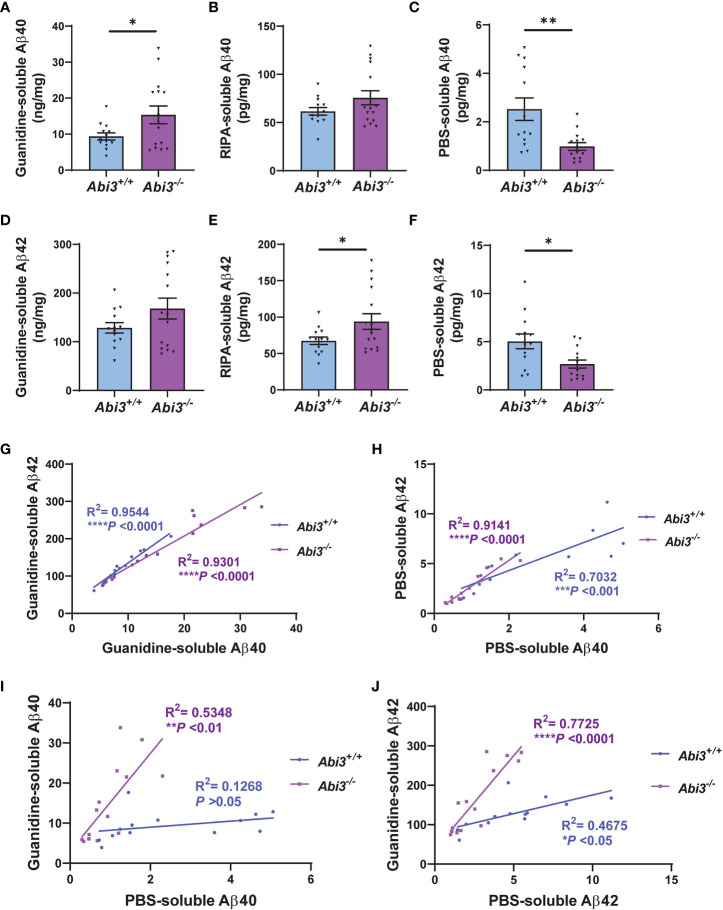
Aβ accumulation increases in *Abi3^-/-^* mice. **(A–C)** Sequential protein extraction was performed on the cortical tissues with PBS, RIPA, and Guanidine buffer. The levels of Aβ40 were measured in the **(A)** guanidine, **(B)** RIPA, and **(C)** PBS-soluble fractions of 5XFAD mouse cortices using an MSD electrochemiluminescence assay. **(D–F)** The levels of Aβ42 were measured in the **(D)** guanidine, **(E)** RIPA, and **(F)** PBS-soluble fractions of 5XFAD mouse cortices (n=13-16). Data represent mean ± SEM. Unpaired two-tailed t-test; *p < 0.05, **p < 0.01. **(G, H)** A significant correlation was observed between **(G)** Guanidine-soluble Aβ40 and Aβ42, and **(H)** PBS-soluble Aβ40 and Aβ42 levels. **(I, J)** Correlation analyses were performed between Guanidine-soluble and PBS-soluble fractions for **(I)** Aβ40 and **(J)** Aβ42 levels. Pearson’s correlation analysis; *p < 0.05, ***p < 0.001, ****p < 0.0001.

To investigate the effect of *Abi3* locus deletion on AD-related pathologies, we bred *Abi3* knock-out mice with the 5XFAD transgenic mouse model of Aβ amyloidosis. We generated 5XFAD mice expressing *Abi3* (*5XFAD;Abi3^+/+^*, referred to as *Abi3^+/+^*) and not expressing *Abi3* (*5XFAD;Abi3^-/-^*, referred to as *Abi3^-/-^*). 5XFAD mice recapitulate many pathological features of AD including Aβ accumulation starting from an early age (28). In our recent study, we reported that Aβ levels were significantly increased in *Abi3^-/-^* mice at 8 months of age, which mimics the late stages of the disease in humans with a significant amount of Aβ accumulation (22). However, it is important to determine the effect of genetic manipulations on disease pathology along the progression of the disease for translational purposes. This is especially critical for microglial targets as many studies demonstrated opposing effects on proteinopathies depending on the disease stage (8–14). Therefore, we used 4.5-month-old *Abi3^+/+^* and *Abi3^-/-^* mice to determine the effect of *Abi3* locus deletion on Aβ accumulation at earlier stages of the disease. We measured the levels of Aβ proteins in the soluble and insoluble fractions of cortical tissues (**Figure 1**). Insoluble, guanidine-extracted, Aβ40 levels were increased 1.6-fold in the cortices of *Abi3^-/-^* mice compared to *Abi3^+/+^* mice (**Figure 1A**). Although there was a trend of increase in *Abi3^-/-^* mice, Aβ40 levels in the RIPA-soluble fraction were not significantly different between the genotypes (**Figure 1B**). On the other hand, PBS-soluble Aβ40 levels were decreased by 61% in *Abi3^-/-^* mice compared to *Abi3^+/+^* mice (**Figure 1C**). Insoluble Aβ42 levels showed a slight increase in *Abi3^-/-^* mice, although it was not significant (**Figure 1D**). RIPA-soluble Aβ42 levels were significantly increased in the *Abi3^-/-^* cohort (**Figure 1E**). Similar to Aβ40, PBS-soluble Aβ42 levels were significantly decreased but with a smaller effect size in *Abi3^-/-^* mice compared to *Abi3^+/+^* mice (**Figure 1F**).

To evaluate the rigor of these biochemical assays, we performed correlation analyses. There was a strong correlation between Aβ40 and Aβ42 levels both in the Guanidine-soluble and PBS-soluble fractions in each genotype (**Figures 1G, H**). Interestingly, the PBS-soluble Aβ40 and Aβ42 levels were decreased in *Abi3^-/-^* mice compared to *Abi3^+/+^* mice (**Figures 1C, F**). This reduction in the PBS-soluble Aβ levels could be simply a secondary consequence of the higher aggregation of Aβ in *Abi3^-/-^* mice, sequestering the PBS-soluble pool of Aβ into the PBS-insoluble (guanidine-soluble) pool. To determine this, we assessed if there was any anti-correlation between the PBS-soluble and the Guanidine-soluble Aβ levels. Aβ40 levels between the PBS-soluble and the Guanidine-soluble fractions did not show any anti-correlation in *Abi3^-/-^* mice (**Figure 1I**). Aβ42 levels also did not show any anti-correlation between these fractions in *Abi3^-/-^* mice (**Figure 1J**). These data suggest that the decrease in the PBS-soluble Aβ40 and Aβ42 levels in *Abi3^-/-^* mice was not due to the sequestering of the PBS-soluble Aβ by higher amyloid levels. Unexpectedly, we found that there was a significant increase in the slope of the linear regression curve for Aβ40 in *Abi3^-/-^* mice compared to *Abi3^+/+^* mice (p<0.001) (**Figure 1I**). A similar increase was also observed in the slope of linear regression curve for Aβ42 levels (p<0.0001) (**Figure 1J**). These findings suggest that the loss of the *Abi3* function may increase the aggregation of Aβ because there is more guanidine-soluble Aβ for the same amount of PBS-soluble Aβ.

### Amyloid plaque load increases in *Abi3* knock-out mice

**Figure 2:**
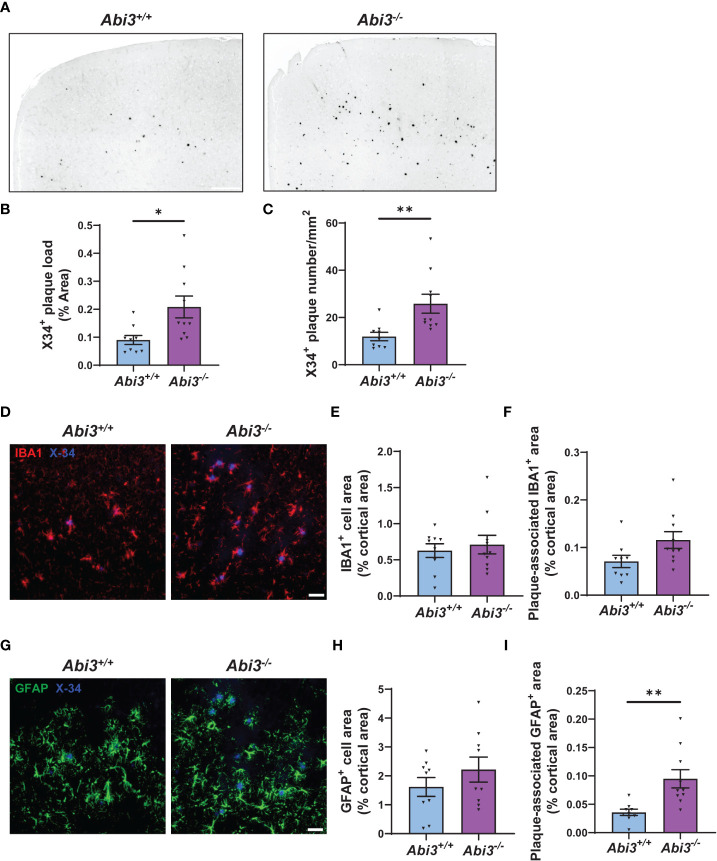
Deletion of the *Abi3* gene locus increases amyloid plaques without altering gliosis in 5XFAD mice. **(A)** Representative images showing coronal brain sections from 4.5-month-old *Abi3^+/+^* and *Abi3^-/-^* mice stained with X34 dye, which detects fibrillar plaques (Scale bar; 250 μm). Black stain indicates X34+ amyloid plaques. **(B)** Quantification of X34+ fibrillar plaque area and **(C)** the number of plaques in mouse cortices. **(D)** Representative images showing coronal brain sections stained with X34 dye and the microglial marker IBA1 antibody (Scale bar; 50 μm). **(E)** Quantification of IBA1+ area and **(F)** the area covered by IBA1 staining colocalized with X34+ plaques. **(G)** Representative images showing coronal brain sections stained with X34 dye and the astrocyte marker GFAP antibody (Scale bar; 50 μm). **(H)** The percent of the area covered by GFAP staining was quantified in the cortices of mice. **(I)** Quantification of GFAP+ area colocalized with X34+ plaques. Data represent mean ± SEM (n=9-10). Unpaired two-tailed t-test; *p < 0.05, **p < 0.01. See also **Supplementary Figure 2**.

The accumulation of Aβ peptides has been considered to be a central event in AD (29). These peptides are secreted into the extracellular compartment and aggregate into fibrils (30–32). To analyze the extent of Aβ aggregation using an additional complementary approach, we assessed amyloid plaque deposition in mice by staining the brain sections with X34 dye that detects only fibrillar plaques (**Figure 2A**). We detected a significant increase both in the area and the number of X34+ amyloid plaques in *Abi3^-/-^* mice compared to *Abi3^+/+^* mice (**Figures 2B, C**). Such an increase in amyloid plaque level in *Abi3^-/-^* mice could be due to alterations in the levels of proteins involved in the production or degradation of Aβ peptides. To address this possibility, we first assessed the levels of proteins regulating Aβ production (**Supplementary Figures 1A-D**). The cleavage of APP by BACE-1 generates β-carboxyl-terminal fragment of APP (β-CTF) and soluble APPβ fragments. β-CTF is further cleaved by gamma-secretase and releases Aβ peptides. Therefore, we measured the levels of APP, BACE-1, and β-CTF in the cortices of *Abi3^+/+^* and *Abi3^-/-^* mice by Western blot. There was no significant difference between the genotypes (**Supplementary Figures 1A-D**). A decrease in Aβ-degrading enzymes can also lead to an increase in Aβ accumulation. Therefore, we assessed the levels of proteins involved in Aβ degradation. We did not detect any significant difference in the IDE and NEP, the major Aβ-degrading enzymes, between the genotypes (**Supplementary Figures 1E-G**). These findings suggest that deletion of the *Abi3* locus may not affect the production and degradation of Aβ at an earlier stage of the pathology.

### Gliosis is not altered in *Abi3* knock-out mice

Neuroinflammation is another pathological hallmark of AD. It is mainly driven by microglia and astrocytes in the brain. These cells have critical functions in development, homeostasis, and disease conditions (33). While earlier studies considered gliosis just as a secondary response to Aβ accumulation in AD, recent studies have demonstrated that gliosis starts at very early stages of the disease and may play an important role during disease progression (6, 34).

Changes in the number, morphology, and transcriptome of microglia have been identified in AD brains throughout the different disease states. These changes were associated with beneficial or harmful effects, depending on the disease stage (7, 35, 36). Because *Abi3* is a microglia-enriched gene, we assessed whether deletion of the *Abi3* locus could affect microgliosis at earlier stages of the pathology. We performed IBA1 immunostaining on the brain sections of *Abi3^+/+^* and *Abi3^-/-^* mice to assess the extent of microgliosis (**Figures 2D, E**). Although there were more plaques in *Abi3^-/-^* mice, the IBA1+ area was not significantly different in *Abi3^-/-^* mice compared to *Abi3^+/+^* mice (**Figure 2E**).

Several studies suggest that microglia cluster around Aβ plaques and phagocytose them (37, 38). Impairment in this function can lead to insufficient Aβ clearance and subsequently increased Aβ deposition. Indeed, we found fewer microglia around the plaques in 8-month-old *Abi3^-/-^* mice in our earlier study (22). To assess whether this effect could be a change occurring at an early time point, we analyzed plaque-associated microglia in the brains of 4.5-month-old *Abi3^+/+^* and *Abi3^-/-^* mice (**Figure 2D**). The percent of the area covered by IBA1+ microglia colocalized with X34+ plaques was not significantly different between the genotypes in the young cohort (**Figure 2F**). Consistent with this, the number of IBA1+ microglia processes around the plaques was not different between the genotypes (**Supplementary Figure 2**).

Next, we assessed the changes in the astrocyte population, another major cell type contributing to the inflammatory response in the brain. Astrocyte proliferation and activation have also been demonstrated in AD (39). Importantly, astrocyte reactivity is regulated by microglia-astrocyte crosstalk (40). Therefore, we assessed the effect of *Abi3* locus deletion on astrogliosis. We stained the brain sections with glial fibrillar acidic protein (GFAP) antibody to label astrocytes and compared the GFAP+ area between the genotypes (**Figures 2G, H**). There was no significant difference in GFAP+ area between the genotypes, suggesting that the loss of function of *Abi3* does not affect astrogliosis (**Figure 2H**). Interestingly, more GFAP+ cells colocalized with plaques in *Abi3^-/-^* mice (**Figure 2I**). It warrants further studies to determine whether the increase in plaque-associated astrocyte area is a response to the increased Aβ accumulation or whether there are other non-cell-autonomous mechanisms.

### Transcriptomic changes in *Abi3* knock-out mice

To gain further insight into the potential mechanisms contributing to the phenotype in *Abi3^-/-^* mice, we performed transcriptomic analysis on the cortices of *Abi3^+/+^* and *Abi3^-/-^* mice. We used the nCounter Mouse AD Consortium panel that can detect 760 genes. These genes are involved in 23 different neuronal or glial pathways and associated with LOAD in the Accelerating Medicines Partnership Alzheimer’s Disease Project (AMP-AD) Consortium study.

**Figure 3:**
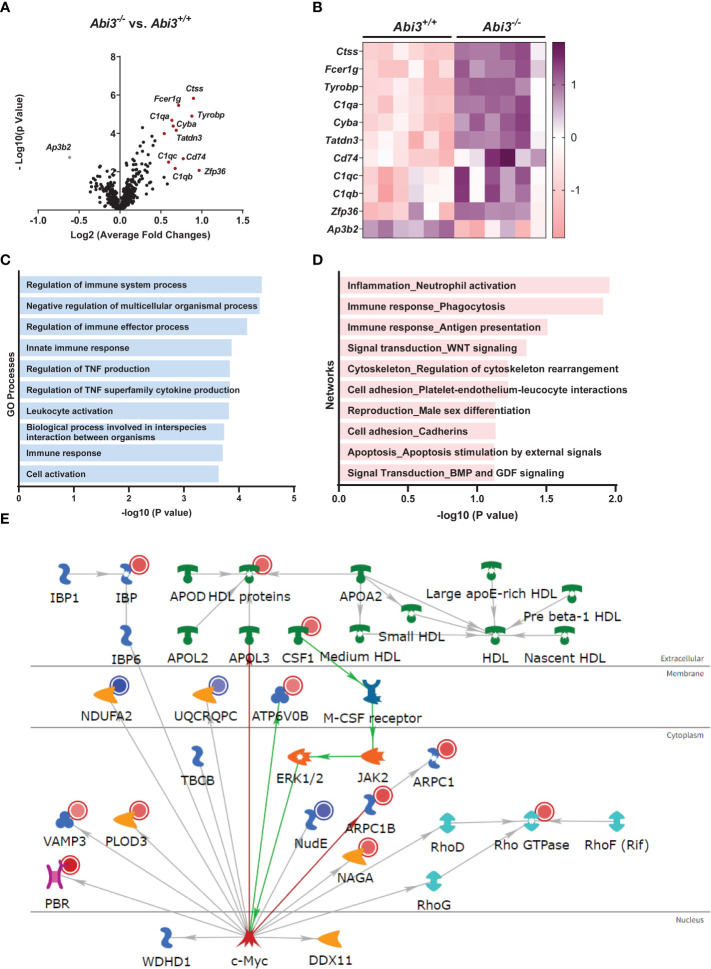
Deletion of the *Abi3* locus alters immune response genes in 5XFAD mice. **(A, B)** Differentially expressed genes (DEGs) were identified in the cortices of 4.5-month-old *Abi3^-/-^* mice compared to *Abi3^+/+^* mice using the nCounter NanoString mouse AD panel (n=6/genotype). **(A)** The Volcano plot demonstrates the fold change (*x*-axis) and statistical significance level expressed as the −log_10_ *P* value (*y*-axis). The red dots represent genes significantly upregulated by more than 1.5-fold in *Abi3^-/-^* mice compared to *Abi3^+/+^* mice. **(B)** The heatmap demonstrates the gene expression levels of DEGs altered by more than 1.5-fold in *Abi3^-/-^* mice compared to *Abi3^+/+^* mice after z-score transformation. Upregulation is shown in purple and downregulation is in pink color. Each column represents each mouse. **(C)** Gene ontology (GO) and **(D)** Network analyses were performed for DEGs using the MetaCore™ software. **(E)** Pathway analysis was performed using MetaCore™. Upregulated genes in our dataset are shown with red circles and downregulated genes are shown with blue circles in the pathmap. Green arrows between nodes represent activation, while grey arrows represent interaction with no specific direction of effect. See also **Supplementary Tables 1**-**3** and **Supplementary Figure 3**.

We determined differentially expressed genes (DEGs) in *Abi3^-/-^* mouse brains compared to *Abi3^+/+^* (**Figure 3A** and **Supplementary Tables 1**, **2**). Among these, *Ctss*, *Fcer1g*, *Tyrobp*, *C1qa*, and *Cyba* were the most significantly upregulated genes in *Abi3^-/-^* mice (**Figures 3A, B**). Importantly, these genes are primarily expressed in microglia and identified in the immune/microglia module in LOAD patient brains (41). Interestingly, the most significantly down-regulated gene, *Ap3b2*, is a neuron-specific gene and is involved in the formation of synaptic vesicles, transfer of membrane proteins to lysosomes, and endocytosis (42, 43). To better understand the biological processes that are regulated by DEGs in *Abi3^-/-^* mice, we performed gene ontology (GO) analysis using the MetaCore™ software. We identified that these DEGs were enriched in many immune response-related biological processes (**Figure 3C**). Network analysis also supported these findings by identifying the “Inflammation_Neutrophil activation” and “Immune response_Phagocytosis” as significant process networks that the DEGs are involved in (**Figure 3D** and **Supplementary Table 3**). We generated a pathmap using the networks that were prioritized based on the number of genes in the canonical pathways identified in our dataset (**Figure 3E**). This also demonstrates the involvement of cytoskeleton networks (*Arpc1, Arpc1b, Rho GTPase*), which are directly relevant to the function of *Abi3*, since ABI3 participates in an actin-regulatory complex (**Figure 3E** and **Supplementary Table 3**). These analyses provide insight into how certain biological processes can be regulated by the interaction of multiple molecular networks that are involved in different cellular pathways. Therefore, alterations in any of these genes may affect the interactions of these cellular pathways, which can collectively result in greater effects in complex diseases.

We also compared the transcriptomic changes in the young cohort with our previous study, where we used the nCounter Mouse AD panel with 8-month-old mice (22) (**Supplementary Figure 3** and **Supplementary Table 2**). Many genetic and functional studies have demonstrated that microglia have a complex role in AD. Their functional and phenotypic diversity over the course of pathology is accompanied by transcriptomic changes identified in human brains and mouse models of AD at different ages using transcriptomic approaches (44–46). While microglia transcriptome is associated with a homeostatic state at earlier stages of the disease, they show a gradual transition into a disease-associated state with a unique transcriptomic signature (44). To investigate the transcriptomic changes during the progression of the Aβ pathology in *Abi3^-/-^* mice, we compared the DEGs between the young (4.5-month-old) and old cohorts (8-month-old) (**Supplementary Figure 3** and **Supplementary Table 2**). We identified 32 common DEGs, 53 young cohort-specific, and 64 old cohort-specific DEGs (**Supplementary Figure 3A**). To identify the shared pathways that were altered during the progression of the pathology in *Abi3^-/-^* mice, we performed pathway analysis with the 32 overlapping DEGs in the young and old cohorts (**Supplementary Figures 3B, C**). GO analysis demonstrated that these common genes were involved in biological processes related to the immune response (**Supplementary Figure 3B**). Network analysis further strengthened the role of *Abi3* in inflammation by identifying the involvement of DEGs in immune response, signal transduction, and apoptosis (**Supplementary Figure 3C**).

In addition to the shared pathways between the young and old cohorts, we aimed to identify the pathways regulated by DEGs unique to young or old cohorts. Interestingly, GO analyses revealed a marked divergence in biological processes that are regulated by young or old cohort-specific DEGs (**Supplementary Figures 3D, E**). Young cohort-specific DEGs are enriched in Rho and Ras signal transduction, and cytoskeleton-related biological processes (**Supplementary Figure 3D**). However, the DEGs specific to the old cohort are involved in oxidative stress-induced apoptotic signaling, immune response, and response to oxidative stress processes (**Supplementary Figure 3E**). These data may support the notion that microglia switch to a more proinflammatory state in the later stages of the disease, which can exacerbate the pathology.

### Alterations in cytokine levels in *Abi3* knock-out mice

**Figure 4:**
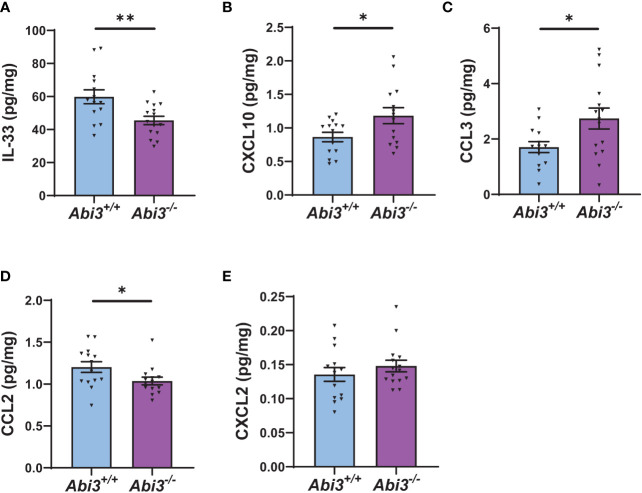
Deletion of the *Abi3* locus alters the levels of secreted cytokines in 5XFAD mice. Protein levels of cytokines were measured in the PBS fraction of 4.5-month-old 5XFAD mouse cortices using the MSD mouse cytokine panel. **(A)** IL-33, **(B)** CXCL10, and **(C)** CCL3 were significantly increased, whereas **(D)** CCL2 was decreased in *Abi3^-/-^* mice compared to *Abi3^+/+^* mice. **(E)** CXCL2 levels did not change between the genotypes. All data were normalized by total protein level and given as mean ± SEM (n=14-15). Unpaired two-tailed t-test, *p < 0.05, **p < 0.01.

Because transcriptomic analyses identified enrichment of immune response in *Abi3^-/-^* mice, we measured the protein levels of secreted and intracellular cytokines in *Abi3^+/+^* and *Abi3^-/-^* mouse brains (**Figure 4** and **Supplementary Figure 4**). We used a mouse cytokine panel and were able to reliably detect 5 out of 9 cytokines in brain samples: interleukin-33 (IL-33), CXCL10, CCL2, CCL3, and CXCL2 (**Figure 4** and **Supplementary Figure 4**). Among these, IL-33 and CCL2 were significantly decreased, whereas CXCL10 and CCL3 were significantly increased in the PBS-soluble fraction of *Abi3^-/-^* mouse cortices compared to *Abi3^+/+^* mice (**Figures 4A-D**). Importantly, we also found a significant increase in the levels of secreted CXCL10 and CCL3 in the old cohort *Abi3^-/-^* mouse cortices in our prior study, strengthening the regulatory role of ABI3 on these cytokines (22). Secreted CXCL2 levels did not show a difference between the genotypes (**Figure 4E**). In the RIPA-soluble (intracellular) fraction, CXCL10, CCL3, and CCL2 were significantly increased in *Abi3^-/-^* mouse cortices compared to *Abi3^+/+^* mice (**Supplementary Figure 4**). These findings suggest that the loss of ABI3 function can trigger an inflammatory response starting from the earlier stages of AD, which can accelerate the progression of the disease.

## Discussion

In this study, we investigated the effect of *Abi3* locus deletion on AD-related pathologies using 4.5-month-old 5XFAD mice to better understand the role of ABI3 in disease progression. In our earlier study, we used 8-month-old 5XFAD mice that mimic the later stages of Aβ pathology (22). In that study, we found a significant increase in Aβ levels and amyloid plaques, accompanied by increased neuroinflammation in 8-month-old *Abi3^-/-^* mice. Earlier studies on other microglial AD risk genes reported opposing effects on AD-related pathologies using various animal models at different ages (8–14). For example, while *Trem2* deficiency increased amyloid plaque load at 6-7-month-old PS2APP transgenic mice, it decreased amyloid deposition at 12 months of age (8). In the APP/PS1 mouse model, *Trem2* deletion reduced Aβ accumulation in 4-month-old mice (10). In another study using the APP/PS1 mouse model, *Trem2* deletion reduced plaque load at 2 months of age, whereas the plaque load was increased in 8-month-old mice (9). These examples can be extended to other microglial genes and other pathological features of AD, such as tau pathology (11–14). All of these studies point to potential distinct functions of microglial genes and their impact on AD pathology depending on the disease stage. Another important lesson from these studies is the need to use different mouse models harboring different AD risk mutations to better understand the role of these genes in AD. Importantly, a recent study using the TgCRND8 mouse model demonstrated that deletion of the *Abi3* locus reduced insoluble Aβ levels and amyloid plaque load in 3-month-old mice, whereas these effects were diminished as mice aged (23). At 6 months of age, insoluble Aβ levels were not different between the genotypes, but *Abi3^-/-^* mice had less amyloid plaque load. These seemingly conflicting results with our data might be due to the different familial AD mutations that the transgenic mice are harboring. Moreover, this study also supports the notion that microglial genes may have divergent effects on the pathologies during disease progression. Therefore, we generated a 4.5-month-old cohort using the 5XFAD transgenic model to gain better insight into the role of *Abi3* during Aβ-amyloidosis progression.

Here, we demonstrated that deletion of the *Abi3* locus significantly increased insoluble Aβ levels in 4.5-month-old 5XFAD mice, similar to what we have found with the old cohort (22). Consistent with the biochemical data, we found an increase in the amyloid plaque load in 4.5-month-old *Abi3^-/-^* mice (**Supplementary Figure 5**). These findings suggest that deletion of the *Abi3* locus decreases the solubility of Aβ isoforms and makes them more prone to aggregate at earlier stages of the pathology in the 5XFAD model. This is further supported by the increased slope of the insoluble-soluble Aβ correlation curve in the *Abi3^-/-^* cohort compared to *Abi3^+/+^*.

In AD, Aβ accumulation is usually accompanied by microgliosis. Although there was an increase in amyloid plaque load in *Abi3^-/-^* mice, we did not detect any increase in microgliosis at 4.5 months of age. In our earlier study, we demonstrated that *Abi3* deficiency impaired the migratory function of microglia *in vitro* and proposed the impairment of migration as one of the key mechanisms by which *Abi3* locus deletion increased Aβ accumulation (22). Consistent with this hypothesis, we detected fewer microglia around the plaques in the old cohort (22). However, in the young cohort, colocalization of microglia with Aβ plaques was not different between *Abi3^+/+^* and *Abi3^-/-^* mice. Our data from young and old cohorts suggest that the migration of microglia may be impaired over time, contributing to the further Aβ accumulation in *Abi3^-/-^* mice later. Since we did not detect an increase in microgliosis corresponding to the increased Aβ plaque load in *Abi3^-/-^* mice, it is also possible that insufficient microgliosis at the early stage of amyloid aggregation might contribute to the increase in Aβ plaque load in *Abi3^-/-^* mice due to inadequate clearance of Aβ by microglia. Similar to our findings, deletion of the *Abi3* locus in the TgCRND8 mouse model did not alter IBA1 and GFAP-positive area in the homozygous knock-out mice (23). Another study reported an increase in the number of IBA1-positive cells, although the total area covered by IBA1 staining was decreased in *Abi3^-/-^* mice without amyloid pathology (47). Taken together, these findings suggest that *Abi3* may have biological context-dependent effects on microgliosis and astrogliosis. It is also noteworthy that these effects might be, in part, due to the alterations in other genes in this mouse model. As we and others demonstrated in prior studies (22, 23), several genes were significantly downregulated in the *Abi3^-/-^* mouse model, including the *Gngt2* and *Gm10039*. These genes or their regulators might be disrupted due to the deletion of the *Abi3* locus.

In our transcriptomic analysis, we have found that DEGs are involved in immune response-related pathways. Importantly, the significantly upregulated genes (*Tyrobp, Ctss, Fcer1g, C1q, Cyba*) were previously identified in the immune/microglia module in LOAD patient brains through an integrative network-based approach (41). *TYROBP* is a key regulator of this gene-network module, driving the expression of other genes. Interestingly, it is also the downstream signaling mediator of several microglial receptors that are implicated in AD, such as TREM2 and CD33 (41, 48). One of the downstream pathways of TREM2-TYROBP is small GTPases in the Ras-homologous (Rho) family (e.g., Cdc42, Rac) (49, 50). Rac signaling is upstream of the ABI-WAVE complex and regulates actin cytoskeleton remodeling (51, 52). The actin cytoskeleton is involved in several critical microglia functions, including surveillance, migration, and phagocytosis. The mechanism by which *Abi3* deficiency increases *Tyrobp* expression warrants further studies. However, these data suggest that *Abi3* deficiency may collectively lead to greater effects due to intersected multiple gene networks.

To gain more insight into the potential pathways that are regulated by *Abi3* during the progression of AD, we compared the DEGs between the 4.5-month-old and 8-month-old cohorts. While the common DEGs between the two cohorts are mostly involved in immune response, we also found distinct pathways regulated by the DEGs that are identified only in the young cohort. These genes are involved in Rho-, Ras-signaling pathways, and cytoskeleton organization, which are directly relevant to the ABI3 function. Interestingly, the old cohort-specific DEGs are enriched in biological processes such as oxidative stress-induced apoptotic signaling and immune response. In summary, *Abi3* deficiency affects key signaling pathways in microglia function and immune response in the young cohort. At later stages, the immune response becomes the dominant biological process in *Abi3^-/-^* mice. Therefore, targeting ABI3 pathway at earlier stages of the pathology may be a desirable approach for the treatment or slowing down the progression of AD.

In addition to the transcriptomic changes, we identified changes in several cytokines at the protein level. Among them, IL-33 was decreased in *Abi3^-/-^* mice. Previously, *IL-33* was identified as an AD risk gene and its level was decreased in the brains of LOAD patients (53). Furthermore, IL-33 administration ameliorated memory deficits and reduced Aβ deposition in the APP/PS1 mouse model (54). It is possible that the reduction in IL-33 level in *Abi3^-/-^* mice might have also contributed to the exacerbated Aβ pathology. We also identified an increase in the levels of secreted CXCL10 and CCL3 in 4.5-month-old *Abi3^-/-^* mice. These cytokines were increased in the old cohort *Abi3^-/-^* mice as well (22). Importantly, CXCL10 and CCL3 were found to be elevated in AD patients and associated with neurological symptoms (55–57). Moreover, both cytokines were shown to impair synaptic functions (58, 59). In fact, we detected synaptic impairment in older *Abi3^-/-^* mice in our earlier study (22). Altogether, these findings suggest that the loss of ABI3 function can cause an inflammatory response starting from earlier stages of AD, which can trigger a chain of toxic events leading to an exacerbated pathology.

In summary, we have demonstrated that deletion of the *Abi3* locus aggravates Aβ pathology in the younger 5XFAD mice, consistent with our prior study using 8-month-old 5XFAD mice. Furthermore, alterations in neuroinflammation pathways start at earlier stages of Aβ pathology in *Abi3^-/-^* mice, which can contribute to the exacerbated pathology in older mice. Additionally, transcriptomic analyses have demonstrated that ABI3 affects multiple AD-associated genes. Alterations in the levels of these genes can also enhance the impact of ABI3 on AD. These data collectively suggest that targeting ABI3 may be a promising therapeutic strategy due to its regulatory effects on multiple key gene networks in AD starting from earlier stages of the disease.

## Data availability statement

The original contributions presented in the study are included in the article/**Supplementary Material**, further inquiries can be directed to the corresponding author/s.

## Ethics statement

The animal study was reviewed and approved by Indiana University School of Medicine Institutional Animal Care and Use Committee.

## Author contributions

HK and JK conceived the study and designed the experiments. HK, DS, BK, BC, and SJ performed the experiments. HK, BK, BC, JM, MA-A, and LD analyzed the data. HK and JK interpreted the results and wrote the manuscript. All authors contributed to the article and approved the submitted version.
