# Supplementary material for: The effect of Abi3 locus deletion on the progression of Alzheimer’s disease-related pathologies
Source: Front Immunol. 2023 Feb 21;14:1102530. doi: 10.3389/fimmu.2023.1102530 (PMC9988916; doi:10.3389/fimmu.2023.1102530)
Supplement: Supplementary file 1 [file DataSheet_1.docx]

Supplementary Figures

**Supplementary Figure 1.** **Effect of *Abi3* locus deletion on the proteins involved in Aβ synthesis and degradation.** Protein levels in the RIPA fraction of the cortices were determined by Western blot. **(A)** Representative images of the blots. Quantification of **(B)** APP, **(C)** BACE1, and **(D)** β-CTF immunoblots. **(E)** Representative images of the blots. Quantification of **(F)** IDE and **(G)** NEP immunoblots. Data were normalized by β-actin levels and expressed as fold-change relative to *Abi3^+/+^* (n=6/genotype). Data represent mean ± SEM. Unpaired two-tailed t-test; p >0.05.

**Supplementary Figure 2. The effect of *Abi3* locus deletion on plaque-associated microglia in 5XFAD mice.** The number of IBA1+ cell processes overlapping with X34+ plaques was quantified based on the area of each plaque. There was no significant difference in the number of plaque-associated IBA1+ processes in the cortices of 4.5-month-old *Abi3^-/-^* mice compared to *Abi3^+/+^* mice. Data represent mean ± SEM (n=9-10). One-way ANOVA and Tukey’s multiple comparison test; p >0.05 (Related to Fig. 2).

**Supplementary Figure 3. Comparison of transcriptomic changes in *Abi3^-/-^* mice relative to *Abi3^+/+^* mice between 4.5 and 8-month-old cohorts. (A)** Differentially expressed genes (DEGs) in *Abi3^-/-^* mice compared to *Abi3^+/+^* mice were identified in the cortices of 4.5- and 8-month-old mice using the nCounter NanoString mouse AD panel (n=6/genotype). There were 32 common, 53 young cohort-specific, and 64 old cohort-specific DEGs. **(B)** Gene ontology and **(C)** Network analyses were performed with 32 common DEGs between young and old cohorts using the MetaCore™ software. These common genes were enriched in immune response-related pathways. Gene ontology analyses were performed using **(D)** young cohort-specific and **(E)** old cohort-specific DEGs. **(D)** The young cohort-specific DEGs were involved in Rho-, Ras-signaling, and cytoskeleton organization. **(E)** The old cohort-specific DEGs were enriched in apoptotic signaling and immune response-related biological processes.

**Supplementary Figure 4. Deletion of the *Abi3* locus alters the levels of intracellular cytokines in 5XFAD mice.** Protein levels of cytokines were measured from the RIPA fraction of 4.5-month-old 5XFAD mouse cortices using the MSD mouse cytokine panel. **(A)** IL-33 levels did not change between the two genotypes. **(B)** CXCL10, **(C)** CCL3, and **(D)** CCL2 were significantly increased in *Abi3^-/-^* mice compared to *Abi3^+/+^* mice. **(E)** CXCL2 levels did not change between the genotypes. All data were normalized by total protein level and given as mean ± SEM. Unpaired two-tailed t-test; ***p*<0.01, *****p*<0.0001 (*Abi3^+/+^*, n=14; *Abi3^-/-^*, n=15).


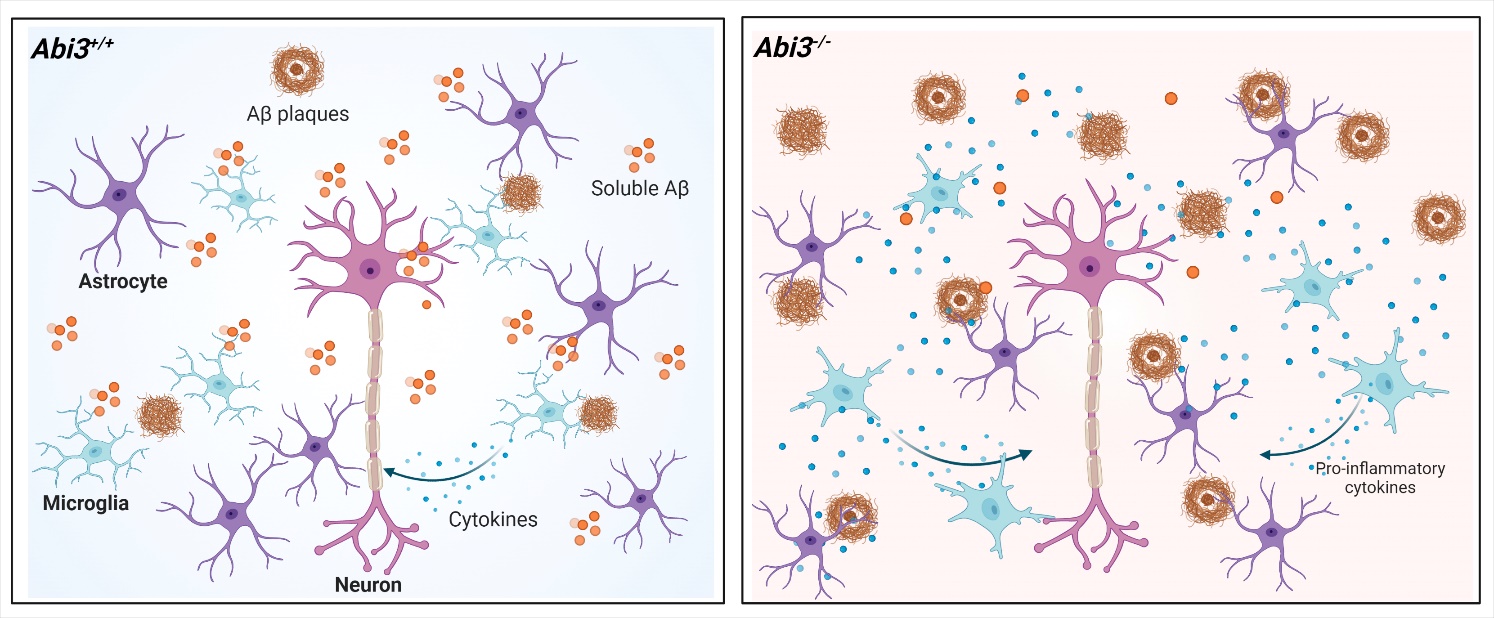


**Supplementary Figure 5. The effect of *Abi3* locus deletion on AD-related pathologies.** Schematic diagram representing the effects of *Abi3* locus deletion on AD-related pathologies in the brains of 4.5-month-old 5XFAD mice. Created with BioRender.com
