# Supplementary material for: The effect of Abi3 locus deletion on the progression of Alzheimer’s disease-related pathologies
Source: Front Immunol. 2023 Feb 21;14:1102530. doi: 10.3389/fimmu.2023.1102530 (PMC9988916; doi:10.3389/fimmu.2023.1102530)
Supplement: Supplementary file 4 [file Table_3.docx]

**Supplementary Table 3.** **Network analysis of the differentially expressed genes in *Abi3^-/-^* mice.** Differentially expressed genes (DEGs) were identified in the cortices of 4.5-month-old *Abi3^-/-^* mice compared to *Abi3^+/+^* mice using nCounter NanoString mouse AD panel (n=6/genotype). Network analysis was performed using MetaCore™. Networks enriched in *Abi3^-/-^* mice and the genes that are differentially regulated in each network are listed in the table.

| **Networks** | **P-value** | **Network objects** |
| --- | --- | --- |
| Inflammation _ Neutrophil activation | 1.107E-02 | CD63, PKC-β, PKC-β2, TNF-R1, p22-phox (CYBA), PREX1 |
| Immune response _ Phagocytosis | 1.227E-02 | APOE, CD63, MSN (moesin), PKC-β, PKC-β1, p22-phox (CYBA), Fc epsilon RI gamma |
| Immune response _ Antigen presentation | 3.100E-02 | Cathepsin S, TNF-R1, CD74, Fc epsilon RI gamma |
| Signal transduction _ WNT signaling | 4.403E-02 | TGF-β receptor type II, PKC-β, PKC-β2, TGF-β receptor type I |
| Cytoskeleton _ Regulation of cytoskeleton rearrangement | 5.971E-02 | MSN (moesin), RhoGDI-β, PREX1, ARPC1B |
| Cell adhesion _ Platelet-endothelium-leucocyte interactions | 5.971E-02 | Protein S, TGF-β receptor type II, CD81, Fc epsilon RI gamma |
| Reproduction _ Male sex differentiation | 7.356E-02 | TGF-β receptor type II, SOX9, PKC-β, BI-1, TGF-β receptor type I |
| Cell adhesion _ Cadherins | 7.356E-02 | ACP1, PKC-β, PKC-β2, PKC-β1, FAT1 |
| Apoptosis _ Apoptosis stimulation by external signals | 7.530E-02 | TGF-β receptor type II, TNF-R1, TGF-β receptor type I |
| Signal Transduction_BMP and GDF signaling | 7.530E-02 | TGF-β receptor type II, SOX9, TGF-β receptor type I |
